# A Biphasic Innate Immune MAPK Response Discriminates between the Yeast and Hyphal Forms of *Candida albicans* in Epithelial Cells

**DOI:** 10.1016/j.chom.2010.08.002

**Published:** 2010-09-16

**Authors:** David L. Moyes, Manohursingh Runglall, Celia Murciano, Chengguo Shen, Deepa Nayar, Selvam Thavaraj, Arinder Kohli, Ayesha Islam, Hector Mora-Montes, Stephen J. Challacombe, Julian R. Naglik

**Affiliations:** 1 Department of Oral Medicine, Pathology, and Immunology, King's College London Dental Institute, King's College London, London SE1 9RT, UK; 2 School of Medical Sciences, Institute of Medical Sciences, University of Aberdeen, Aberdeen AB25 2ZD, UK

## Abstract

Discriminating between commensal and pathogenic states of opportunistic pathogens is critical for host mucosal defense and homeostasis. The opportunistic human fungal pathogen *Candida albicans* is also a constituent of the normal oral flora and grows either as yeasts or hyphae. We demonstrate that oral epithelial cells orchestrate an innate response to *C. albicans* via NF-κB and a biphasic MAPK response. Activation of NF-κB and the first MAPK phase, constituting c-Jun activation, is independent of morphology and due to fungal cell wall recognition. Activation of the second MAPK phase, constituting MKP1 and c-Fos activation, is dependent upon hypha formation and fungal burdens and correlates with proinflammatory responses. Such biphasic response may allow epithelial tissues to remain quiescent under low fungal burdens while responding specifically and strongly to damage-inducing hyphae when burdens increase. MAPK/MKP1/c-Fos activation may represent a “danger response” pathway that is critical for identifying and responding to the pathogenic switch of commensal microbes.

## Highlights

► NF-κB and MAPK control epithelial effector responses against *Candida albicans* ► c-Jun activation is independent of morphology and due to fungal cell wall recognition ► MAPK/MKP-1/c-Fos pathway activation is dependent on fungal hyphae and burdens ► MAPK discriminatory response may dictate *C. albicans* mucosal colonization in vivo

## Introduction

Mucosal epithelia possess distinct mechanisms that enable discrimination between harmless commensal organisms and disease-causing pathogenic organisms. This process results in either nonresponsiveness or homeostasis (commensals) or activation of an immune response (pathogens). Although much attention has been given to bacteria in this context, the same cannot be said for fungal pathogens, which are often clinically more important than their bacterial counterparts. The most significant fungal pathogens of humans are *Candida* species, some of which are polymorphic organisms capable of growing either as yeasts or hyphae. *Candida* species, particularly *C*. *albicans*, are normal constituents of the normal oral and gut flora in ∼50% of the population but can also cause a variety of mucosal diseases with significant morbidity (Odds, 1988). Given that potentially life-threatening systemic complications arise from breaches of the mucosal barrier, it is of paramount importance to understand how epithelial cells recognize and restrict these fungi to mucosal surfaces. Currently, we lack understanding of how *Candida* pathogens interact with human mucosal surfaces or the immune mechanisms that enable epithelial cells to discriminate between the different morphological states of these fungi.

Innate immune responsiveness to pathogens is usually mediated by pattern recognition receptors (PRRs). PRRs recognize specific pathogen-associated molecular patterns (PAMPs), which leads to the activation of intracellular signaling pathways and specific transcription factors to mediate their effects (Kumagai et al., 2008; Medzhitov, 2007). In myeloid cells, several different PRRs have been associated with the recognition of *C. albicans* and other fungi, including mannose receptor (MR), toll-like receptor (TLR)2, TLR4, and dectin-1 (Netea et al., 2006, 2008). Activation of these receptors either individually or in combination leads to an effector response involving secretion of cytokines and other inflammatory mediators, as well as enhanced phagocytosis of pathogens (Kennedy et al., 2007). This process is dependent on several different signaling pathways, including NF-κB and mitogen-activated protein kinase (MAPK), which modulate gene transcription through various transcription factors, including c-Jun (AP-1) and NF-κB (Lee and Kim, 2007). In bacterial infection, stimulation of myeloid cells also leads to the activation of several downstream proteins involved in regulating these signaling pathways, including the MAPK phosphatase MKP1 (Wang and Liu, 2007; Zhao et al., 2006). Likewise, epithelial cells generate immune-based effector responses to different microbes (Abreu et al., 2005); however, little is known with regard to how the epithelium responds to *C. albicans*.

Previously, we identified an immune mechanism in which human epithelial TLR4 could directly protect oral mucosal surfaces from infection with *C. albicans* via a process dependent on polymorphonuclear cells (PMNs) (Weindl et al., 2007). This description of a PMN-dependent TLR4-mediated protective mechanism at epithelial surfaces provided significant insights into how microbial infections might be managed and controlled in the oral mucosa. The key event initiating the whole protective process is the induction of an epithelial proinflammatory response by *C. albicans*. However, the mechanism by which this effector response is initiated is unknown.

In this study, we show that infection of oral epithelial cells with *C. albicans* leads to activation of both the NF-κB and MAPK pathways, which orchestrate the effector response via the production of proinflammatory mediators. The MAPK pathway is activated in a biphasic manner, which involves MKP1 and c-Fos transcription factor, and permits discrimination between the yeast and hyphal forms of *C. albicans*. Furthermore, MAPK/MKP1/c-Fos activation is dependent upon fungal burdens and thus may constitute a “danger response” mechanism alerting the host to when this normally commensal organism has turned into a potentially dangerous invading pathogen.

## Results

### Activation of the NF-κB Pathway

**Figure 1:**
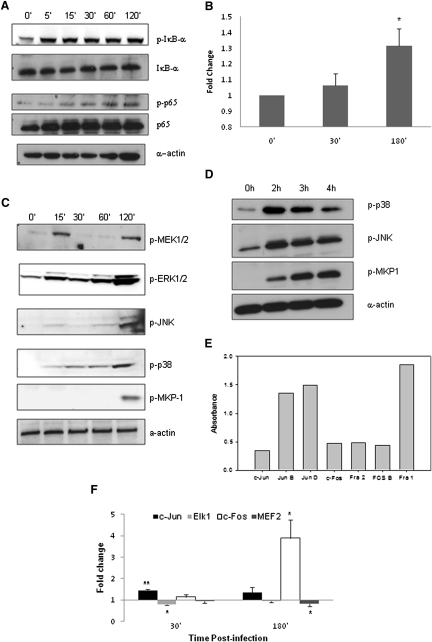
*C. albicans* Infection of TR146 Epithelial Cells Activates NF-κB and MAPK Signaling (A) Increasing phosphorylation of IκB-α and p65 from 5 min postinfection onward. Bands are shown relative to unphosphorylated protein and α-actin loading control. (B) Transcription factor DNA binding activity of p65 after *C. albicans* infection as measured by TransAM ELISA. (C) Increased phosphorylation of MEK1/2, ERK1/2, JNK, and p38 after 15 min postinfection, with further phosphorylation at 2 hr, indicating a biphasic response. Also, phosphorylation of MKP1 only at 2 hr postinfection, indicating stabilization of the MAPK response. (D) Decreasing levels of p38 and JNK phosphorylation after 2 hr infection, matching with increasing levels of MKP1 phosphorylation. (E) Levels of DNA binding activity (absorbance values) of AP-1 transcription factor members in resting TR146 cells. (F) Changes in binding activity of c-Fos, c-Jun, Elk1, and MEF2 after infection; data represented as fold change relative to resting cells. A *C. albicans*:epithelial cell MOI of 10:1 was used. Data are (A, C–E) representative of three independent experiments or mean of at least three (B) or six (F) independent experiments ± SEM. ^∗^p < 0.05, ^∗∗^p < 0.01. (Two bands are seen for ERK1/2 and JNK, as two different proteins make up these complexes). See also Figure S1.

*C. albicans*-induced IκB-α phosphorylation, a key event in NF-κB pathway activation, occurs as early as 5 min postinfection, which persisted for a 2 hr period (Figure 1A). Likewise, phosphorylated p65 protein levels increased steadily during the first 2 hr. Using TransAM transcription factor ELISA to determine the DNA binding activity of all NF-κB proteins (p65, p50, c-Rel, p52, and RelB), we demonstrated that only p65 binding activity increased (mirroring IκB-α phosphorylation) by 3 hr (Figure 1B). These data suggest that NF-κB signaling via p65/p50 heterodimers is the predominant mechanism induced in oral epithelial cells by *C. albicans*.

### Activation of the MAPK Pathway

Unlike NF-κB activation, *C. albicans* induced a biphasic response for the phosphorylation of JNK and p38 and, particularly, ERK1/2 and MEK1/2 (a MAP2K upstream of ERK1/2) (Figure 1C). Phosphorylation increased from resting levels at 15 min and then either subsided (MEK1/2, ERK1/2) or plateaued (p38, JNK). There then followed an increase in phosphorylation for all MAPK proteins at 2 hr. We next explored downstream MAPK-induced events by investigating MKP1 (DUSP1) stabilization. MKP1 is a key phosphatase involved in a negative feedback loop regulating the MAPK pathway (Liu et al., 2007) and is phosphorylated by ERK1/2 to prevent its degradation. This event is independent of gene transcription and is mediated by ERK1/2 (Brondello et al., 1999). Of interest, phosphorylated MKP1 only appeared at 2 hr postinfection, coinciding with the second, stronger MAPK response, providing support for the biphasic response hypothesis (Figure 1C). Infection of OKF6-TERT2 cells (an immortalized oral epithelial line) gave identical kinetics of MKP1 phosphorylation (Figure S1 available online), indicating that this phenomenon is not specific to carcinoma cells. Importantly, commensurate with its function as p38/JNK phosphatase, as levels of phosphorylated MKP1 increase after 2 hr, levels of phosphorylated p38 and JNK also decrease (Figure 1D).

MAPK signaling is associated with AP-1 transcription factor complex activation, which is a heterodimer of a Jun protein (JunB, JunD, and c-Jun) and a Fos protein (Fra1, Fra2, c-Fos, and FosB) (Shaywitz and Greenberg, 1999; Wisdom, 1999). In resting oral cells, DNA binding activity is present for all Jun and Fos proteins (Figure 1E). *C. albicans* infection results in a significant increase in c-Jun binding by 30 min (Figure 1F), which returns to baseline levels by 3 hr. In contrast, c-Fos binding showed no early change but was strongly upregulated at 3 hr postinfection (Figure 1F). The binding activity of other Jun or Fos proteins was unaltered (data not shown). As well as the AP-1 family, we investigated the binding activity of other MAPK-induced transcription factors, including c-Myc, ATF2, Elk-1, and MEF2. Elk-1 binding significantly decreased at 30 min but returned to resting levels by 3 hr, whereas MEF2 binding significantly decreased only after 3 hr (Figure 1F). ATF2 and c-Myc binding activity was unchanged at both time points (data not shown). The data demonstrate that oral epithelial cells direct a specific response to *C. albicans*, whereby opposing changes in transcription factor activation is mirrored at 30 min (c-Jun and Elk-1) and 3 hr (c-Fos and MEF2) postinfection. This differential transcription factor activity parallels the biphasic MAPK response observed at the signal pathway level.

### Functional Role of NF-κB and MAPK Pathways in Inducing Epithelial Effector Responses

To determine the functional role of the NF-κB and MAPK pathways in inducing effector cytokine responses, we inhibited the respective pathways 4 hr prior to infection with *C. albicans*. Inhibition of NF-κB with BAY11-7082 (IκB-α inhibitor) resulted in significantly decreased production of IL-1α (46%), IL-6 (72%), and G-CSF (79%) (Figure S2A). Inhibition of JNK (SP600125) leads to a decrease in G-CSF (41%), IL-6 (15%), IL-1α (54%), and IL-1β (67%) (Figure S2B). Inhibition of p38 (SB203580) results in downregulation of G-CSF (33%), GM-CSF (45%,), IL-1α (43%), and IL-1β (38%) (Figure S2C). Inhibition of ERK1/2 (FR180204) results in a decrease in GM-CSF only (36%) (Figure S2D).

**Figure 2:**
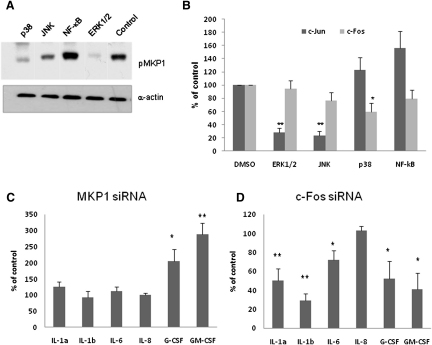
Regulation of MKP1, c-*jun*, c-Fos, and Cytokine Activation (A) Levels of MKP1 phosphorylation 2 hr after *C. albicans* infection (MOI = 10) in TR146 cells after inhibition of different pathways. (B) Level of inhibition of c-Jun (30 min) and c-Fos (3 hr) DNA binding activity after pretreatment for 4 hr with signaling pathway inhibitors. (C and D) Effect of MKP1 and c-Fos knockdown, respectively, on cytokine production (MOI of 0.01). Data are expressed as percentage of vehicle control levels (B–D) and are representative of three independent experiments (A) or are the mean of four independent experiments ± SEM (B–D). ^∗^p < 0.05; ^∗∗^p < 0.01; ^∗∗∗^p < 0.001. See also Figure S2.

As MKP1 and c-Fos activation constituted the second stage of the biphasic MAPK response, we identified the pathways responsible for their activation. Inhibition of NF-κB did not decrease activation of either protein (Figures 2A and 2B). In contrast, inhibition of any MAPK protein, but particularly ERK1/2, resulted in reduced MKP1 phosphorylation (ERK1/2 > p38 > JNK) (Figure 2A), confirming that regulation of MKP1 activity is predominantly by ERK1/2. Likewise, c-Jun activation is induced via the ERK1/2 and JNK pathways (Figure 2B). However, c-Fos activation was predominantly under p38 control (Figure 2B).

We investigated the role of MKP1 and c-Fos following knockdown with siRNAs. MKP1 (Kinney et al., 2008) and c-Fos (Chambellan et al., 2009) siRNAs were quality controlled previously; however, knockdown efficiency was confirmed by qPCR and western blotting, with cell viability after siRNA transfection similar to the viability of control cells (Figure S2E–S2G). Knockdown of MKP1 resulted in a significant increase in G-CSF (206%) and GM-CSF (289%) (Figure 2C), which was expected, given that MKP1 modulates the MAPK pathway by selectively dephosphorylating p38 and JNK. Knockdown of c-Fos resulted in significant reduction in the levels of IL-1α (50%), IL-1β (81%), IL-6 (28%), G-CSF (47%), and GM-CSF (59%) (Figure 2D), suggesting that c-Fos is the main transcription factor controlling MAPK-induced activation of the proinflammatory response. Together, these data indicate that *C. albicans* elicits effector responses in oral epithelial cells via NF-κB and MAPK signaling pathways, with the latter controlling MKP1 and c-Fos activation.

### MKP1, c-Fos, and Cytokine Activation Is Dependent on *C. albicans* Morphology

**Figure 3:**
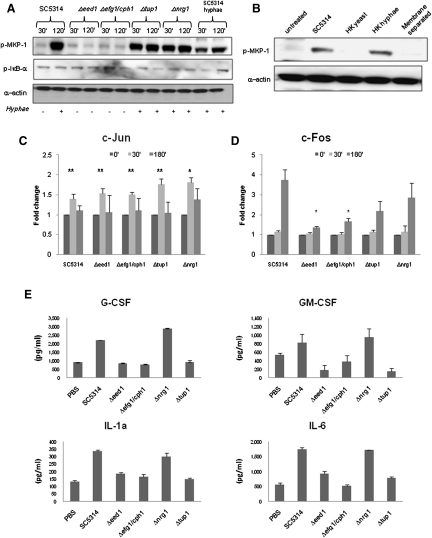
Specific Activation of MKP1 and Transcription Factors and Cytokine Induction by *C. albicans* (A) Immunoblot of phosphorylated MKP1 and IκB-α after infection of TR146 cells with wild-type (SC5314), hyperfilamentous (*Δtup1* and *Δnrg1*), and nonfilamentous (*Δeed1* and Δ*efg1/cph1*) strains or pregrown *C. albicans* hyphae. The morphological status of the strains at each time point is indicated below the immunoblots (presence [+] or absence [−] of filaments). (B–E) (B) Induction of MKP1 phosphorylation at 2 hr using heat-killed (HK) wild-type (SC5314) yeast or hyphae or by SC5314 prevented from direct contact with epithelial cells by a 0.4 μm membrane. We also show changes in binding activity of c-Jun (C) and c-Fos (D) at 30 min and 3 hr infection with nonfilamentous (*Δeed1* and *Δefg1/cph1*) or hyperfilamentous (*Δtup1* and *Δnrg1*) mutants. (E) Induction of cytokines after 24 hr infection with nonfilamentous (*Δeed1* and *Δefg1/cph1*) or hyperfilamentous (*Δtup1* and *Δnrg1*) mutants. GM-CSF production appears to decrease, but this was not significant or reproduced with the other cytokines. An MOI of 10 was used for infections in (A–D), and an MOI of 0.01 was used for (E). Data are (A and B) representative of three independent experiments, (C and D) mean of four experiments (± SEM) or (E) representative of three experiments. ^∗^p < 0.05; ^∗∗^p < 0.01. See also Figure S3.

*C. albicans* infection of oral epithelial cells results in yeast-to-hyphal transition within the first hour. Given the important role of hypha formation in mucosal pathogenicity (Naglik et al., 2006, 2008; Zakikhany et al., 2007), we hypothesized that the second stage (2 hr) of the biphasic MAPK response, constituting MKP1 and c-Fos activation, might be due to a specific response to filamentation. To test this, we infected TR146 cells with wild-type *C. albicans* (SC5314) and four mutants—*Δefg1/cph1* (unable to form hyphae), *Δeed1* (severely restricted filament formation in the first ∼3 hr, after which reverts back to the yeast phase), *Δtup1* (hyperfilamentous but locked in the pseudohyphal phase), and *Δnrg1* (hyperfilamentous hyphae) —for 30 min and 2 hr and tested for MKP1 phosphorylation and c-Jun and c-Fos binding. The morphology of these mutants at 30 min and 2 hr is shown in Figure S3. Only the hyperfilamentous mutants (*Δtup1* and *Δnrg1*) induced MKP1 phosphorylation at 30 min and 2 hr, whereas neither of the nonfilamentous mutants (*Δeed1* and *Δefg1/cph1*) induced MKP1 phosphorylation at either time point (Figure 3A) (or at 3 hr postinfection; Figure S3). As previously, SC5314 wild-type induced MKP1 phosphorylation only at 2 hr when hypha formation was evident. When wild-type cells were preinduced to form germ tubes for 3 hr prior to epithelial infection (SC5314 hyphae), MKP1 phosphorylation was observed at 30 min as well 2 hr (Figure 3A). There was no difference between the strains in the kinetics of IκB-α phosphorylation (Figure 3A), indicating that NF-κB activation is independent of morphology. Physical separation of *C. albicans* from epithelial cells with a porous membrane (0.4 μm) resulted in a complete loss of MKP1 phosphorylation, indicating that MKP1 activation is not a result of secreted factors but is contact dependent (Figure 3B). MKP1 phosphorylation was also induced by heat-killed hyphae, but not heat-killed yeast, indicating that MKP1 activation is hypha dependent but does not necessarily require viability (Figure 3B). The data indicate that MKP1 phosphorylation is the result of contact-dependent hypha recognition.

We next investigated c-Jun and c-Fos activation to determine whether the hypha-associated biphasic MAPK response was mirrored at the transcription factor level. All four mutants activated c-Jun to a similar degree, indicating that activation of the first MAPK phase was independent of morphology (Figure 3C). In contrast, compared with the wild-type strain (SC5314), there was a significant reduction in c-Fos activity in the *Δeed1* and *Δefg1/cph1* nonfilamentous mutants, but not the hyperfilamentous *Δtup1* and *Δnrg1* mutants (Figure 3D), although neither *Δtup1* nor *Δnrg1* induced c-Fos activity as high as the wild-type. The reduced levels of c-Fos activation at 3 hr with the two hyperfilamentous mutants can be explained partly by their reduced level of adherence (Figure S3) and partly by the lack of fungal-epithelial cell contact (i.e., the wild-type yeast cells settled rapidly onto the epithelial surface prior to hypha formation, whereas the hyperfilamentous strains “floated” for a prolonged time period before gradually settling, thus reducing the multiplicity of infection [MOI] and threshold level of activation). Furthermore, both heat-killed hyphae and yeast activated c-Jun, whereas only heat-killed hyphae activated c-Fos, albeit to a lesser degree than viable hyphae (data not shown). This indicates that, although viability and cell damage induced by hyphae is not essential to induce c-Fos activity, viability further activates c-Fos, and the presence of the filamentous form is required. Finally, neither of the nonfilamentous mutants induced cytokine production (Figure 3E). Of interest, of the hyperfilamentous mutants, only *Δnrg1* and not *Δtup1* induced cytokines, suggesting that cytokine production is, in part, independent of MAPK-induced c-Fos activation (i.e., probably NF-κB mediated). Treatment of cells with heat-killed yeast or hyphae failed to induce cytokine secretion at an MOI of 10 after 24 hr (data not shown).

Given that *Δtup1* is thought to be locked in the pseudohyphal phase but still activated MKP1, we tested whether *Δtup1* expresses hypha-specific marker genes. Near identical expression levels of *ECE1* and *HYR1* were observed in *Δtup1*, *Δnrg1*, and SC5314 wild-type after 2 hr growth in cell culture medium (same time points used to assess MKP1 and c-Fos activation) as compared with the nonfilamentous mutants *Δeed1* and *Δefg1/cph1* (data not shown). This suggests that *Δtup1* may activate MKP1 via similar surface moieties as *Δnrg1* and SC5314 wild-type hyphae, even though *Δtup1* is locked in the pseudohyphal phase. We conclude that MKP1 is activated through epithelial recognition of moieties associated with *C. albicans* filamentation.

### MKP1, c-Fos, and Cytokine Activation Is Dependent on Fungal Burden

**Figure 4:**
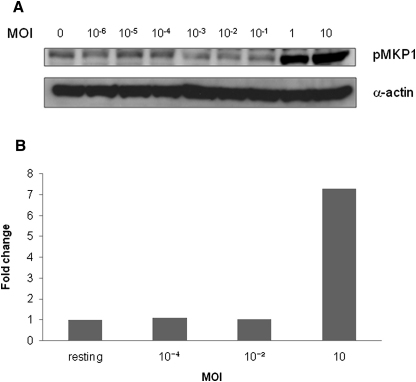
Effect of Fungal Burdens on MKP1 and c-Fos Activation (A and B) Induction of (A) MKP1 phosphorylation and (B) c-Fos DNA binding activity by differing fungal burdens. An MOI of 1 or higher is required to activate the second MAPK phase. Wild-type SC5314 *C. albicans* was added as yeast cells at each MOI, and infections were left for (A) 2 hr or (B) 3 hr in each case. Data are representative of three independent experiments.

Clinically, oral candidiasis is associated with fungal burdens two or three logs higher than that observed during colonization (Naglik et al., 1999, 2003). To determine whether induction of MKP1, c-Fos, and cytokines required a threshold level of activation, we stimulated TR146 cells with different doses of *C. albicans* SC5314 (10^1^–10^7^ cells/ml), equivalent to a MOI of 0.00001–10 *C. albicans* cells per epithelial cell. MKP1 and c-Fos were only activated at an MOI of 1 or above, indicating that a threshold level of activation is required and that each epithelial cell is able to recognize and discriminate against a single fungal cell (yeast or hyphal) (Figures 4A and 4B).

### The MAPK Discriminatory Response is *Candida* Specific but Independent of Structural Cell Wall Polysaccharides and Adherence

**Figure 5:**
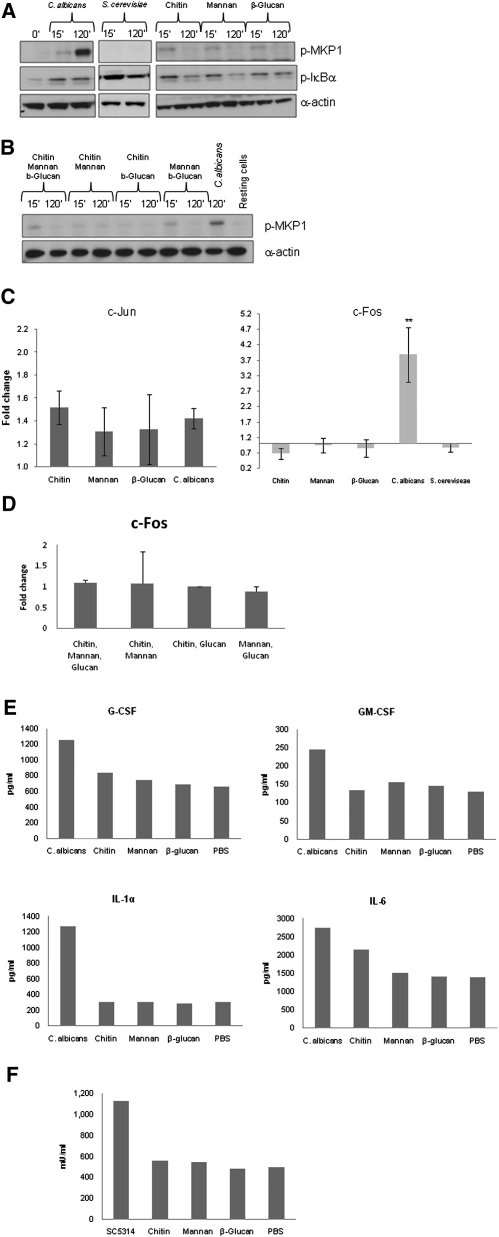
Activation of MKP1 and c-Fos by *C. albicans* and *S. cerevisiae* and Epithelial Responses to Fungal Cell Wall Components (A–D) (A and B) Phosphorylation of MKP1 and IκBα after 2 hr or (C and D) activation of c-Jun and c-Fos DNA binding activity after 30 min and 3 hr of infection, respectively, with either *C. albicans* or *S. cerevisiae* or treatment with 2 μg/ml *C. albicans* chitin, 50 μg/ml *C. albicans N-* and *O-* mannan, or 100 μg/ml β-glucan microspheres (individually or in combination) for the same time periods. Only *C. albicans* activates MKP1 and c-Fos. (C and D) Data are ± SEM. (E) Moiety-induced cytokine production after 24 hr as measured by multiplex microbead assay. (F) Moiety-induced damage as measured by LDH release after 24 hr. Doses for (B)–(D) were the same as those for (A). All experiments are (A and B) representative of or (C–E) the mean of at least three independent experiments. An MOI of 10 was used for both species. ^∗^p < 0.05; ^∗∗^p < 0.01. See also Figure S4.

Given the important role of hypha formation in inducing the MAPK biphasic response by *C. albicans*, we investigated whether similar responses were induced by the nonpathogenic fungus *S. cerevisiae*, which does not produce hyphae in our system. Only *C. albicans* induced MKP1 phosphorylation and c-Fos activation (Figure 5A and 5B) at an MOI of 10, indicating that MKP1 and c-Fos activation is associated specifically with responses to pathogenic fungi. IκBα phosphorylation was similar between the two fungal species (Figure 5A), indicating that NF-κB activation is not only independent of morphology, but also likely to be a general response to fungi.

Because *S. cerevisiae* and *C*. *albicans* yeast and hyphae share common structural components, we next determined whether specific cell wall polysaccharides were able to activate the discriminatory mechanism. Stimulation of TR146 cells with purified *C. albicans N-* and *O-*mannans, chitin, and β-glucan microparticles (gift from David Williams) resulted in activation of NF-κB (IκB-α) (Figure 5A) and the first MAPK response (30 min: c-Jun) (Figure 5C). However, none of these fungal components activated the second MAPK response (MKP1/c-Fos), cytokine production, or cell damage (Figures 5A, 5C, 5E, and 5F). Combinations of these structural moieties were also unable to activate MKP1 or c-Fos (Figures 5B and 5D). This demonstrates that fungal cell wall polysaccharides are unlikely to be responsible for activating either the proinflammatory response or the filamentation-associated discriminatory mechanism.

*C*. *albicans* hyphae adhere better to many surfaces than yeast cells. However, activation of MKP1 or c-Fos was independent of the ability of yeast and hyphal forms to adhere (Figure S3). Neither of the nonfilamentous mutants (Δ*eed1* and Δ*efg1/cph1*) activated MKP1 and c-Fos, but Δ*eed1* adhered as well as the wild-type strain (SC5314). Likewise, both the hyperfilamentous mutants (Δ*nrg1* and Δ*tup1*) activate MKP1 and c-Fos, but Δ*nrg1* adhered better than Δ*tup1*. Finally, a clinical strain (*C. albicans* 529L) that does not produce true hyphae (see later) adhered to TR146 cells to the same degree as SC5314 (hyphal producer), yet only SC5314 activated MKP1 and c-Fos.

### The MAPK Discriminatory Response and Receptor Induction

TLR2, TLR4, MR, and dectin-1 are the PRRs known to initiate antifungal responses in myeloid cells (Netea et al., 2008). As shown earlier, neither β-glucan nor mannan (the ligands for dectin-1 and MR, respectively) induced epithelial cytokine responses. Also, although present, SYK was not phosphorylated by *C. albicans* (Figure S4), and thus dectin-1/SYK signaling is not induced. These data indicate that dectin-1 and MR are unlikely mediators of the *C*. *albicans* discriminatory response. Additional blocking of TLR2 and TLR4 activation using neutralizing antibodies also indicated no role for these receptors in c-Fos activation (Figure S7). Furthermore, siRNA knockdown of TLR2 and TLR4 showed no alterations in MKP1 phosphorylation compared with control siRNA after *C. albicans* infection (Figure S4). We conclude that the second MAPK response enabling epithelial discrimination between *C. albicans* yeast and filamentous forms is independent of canonical PRR activation and is probably mediated via as yet uncharacterized or potentially unique receptor-based detection mechanisms.

### The MAPK Discriminatory Response May Dictate Whether *C. albicans* Colonizes a Mucosal Surface In Vivo

**Figure 6:**
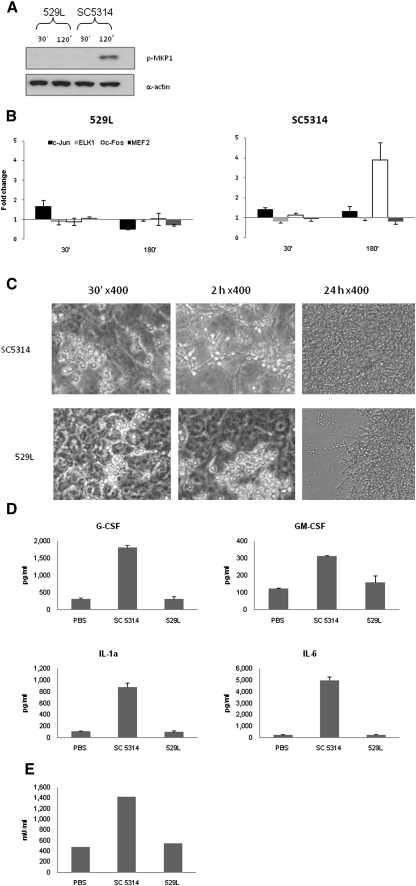
Activation of the Biphasic MAPK Response May Be Required for Colonization in a Murine Model (A and B) (A) Immunoblot analysis of MKP1 phosphorylation and (B) changes in transcription factor binding activity after infection with *C. albicans* SC5314 (noncolonizing) and 529L (colonizing). (C) Morphology of *C. albicans* strains on epithelial cells after 30 min, 2 hr, and 24 hr. (D) Secretion of cytokines after infection with *C. albicans* 529L and SC5314 for 24 hr. An MOI of 10 was used for (A) and (B) and an MOI of 0.01 for (D) and (E). Data are (A and C) representative of three independent experiments or (B, D, and E) mean of three independent experiments ± SEM.

The MAPK discriminatory response was identified using two separate cell lines (TR146 and OKF6-TERT2). We thus sought to confirm in vivo relevance using two approaches. First, previously, we established a murine model of concurrent oral and vaginal *C. albicans* colonization, whereby we identified a differential ability of *C. albicans* strains to colonize both mucosae (Rahman et al., 2007). The best-colonizing *C. albicans* strain 529L and the noncolonizing wild-type strain SC5314 were chosen as representative strains to determine whether the ability to colonize a mucosal surface in vivo may be related to hypha formation and the biphasic MAPK response. Both strains phosphorylated IκB-α equally efficiently (data not shown), but unlike SC5314, 529L was unable to induce MKP1 phosphorylation (Figure 6A) or increase c-Fos binding activity, even though c-Jun, ELK-1, and MEF2 binding activity was identical to SC5314 (Figure 6B). Microscopic examination revealed that 529L was severely restricted in hypha formation in the presence of epithelial cells at 30 min and 2 hr and, after 24 hr, manifested as yeasts and/or pseudohyphae (Figure 6C). Furthermore, unlike SC5314, 529L also failed to induce the production of cytokines (Figure 6D) or to cause damage (Figure 6E) after 24 hr.

**Figure 7:**
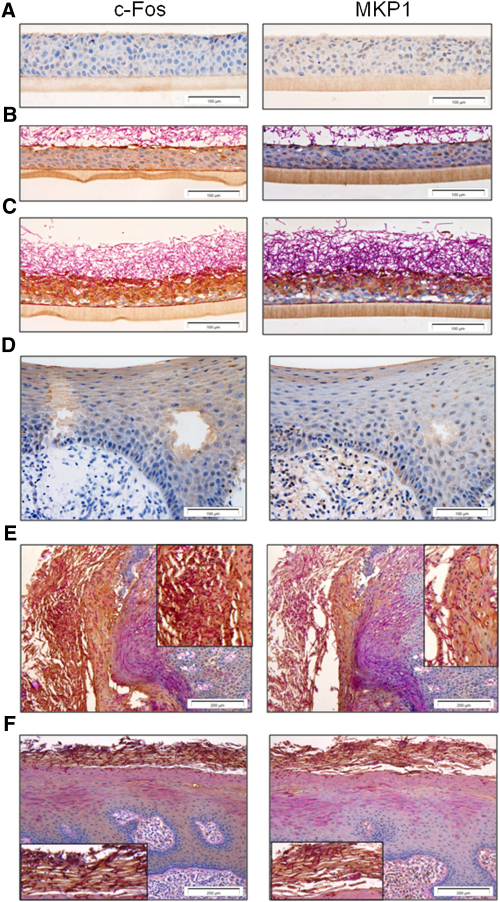
Expression of c-Fos and MKP1 in Oral Epithelium (A–C) (A) Resting expression of c-Fos and MKP1 in oral RHE. Upregulation of c-Fos and MKP1 expression is associated with contact with *Candida* hyphae at (B) the surface after 4 hr and (C) throughout the epithelial layer when hyphae penetrate and invade at 24 hr (dark-brown staining). (D) Resting expression levels of c-Fos and MKP1 in control biopsies of human oral epithelium. (E and F) Increased expression of both c-Fos and MKP1 in two different oral biopsies with *Candida* infection (E, left, and F, top; dark-brown staining). Insets in (E) and (F) show an enlarged view of the region of *Candida* infection in each respective biopsy.

Second, we determined the presence of MKP1 and c-Fos in organotypic models of oral reconstituted human epithelium (oral RHE) and in biopsies from two patients with oral *Candida* infections. In uninfected oral RHE, there was minimal expression of either c-Fos or MKP1 (Figure 7A). However, 4 hr postinfection, surface activation of c-Fos and MKP-1 was evident (dark-brown staining) in regions of hyphal-epithelial cell contact (Figure 7B), and after 24 hr, there was significant hyphal penetration (PAS staining), which correlated with a significant increase in MKP1 and c-Fos expression (Figure 7C). A clear delineation can be observed with cells in contact with hyphae showing expression of both MKP1 and c-Fos and cells in adjacent uninfected areas showing little MKP1 and c-Fos expression.

In control, uninfected human biopsies, c-Fos was expressed at low resting levels but only in the upper layers of the epithelium (and not the underlying connective tissue) (Figure 7D). However, in two separate oral biopsies with *Candida* infection, there was a distinctly visible increase in both MKP1 and c-Fos expression (Figures 7E and 7F; dark-brown staining), demonstrating that MKP1 and c-Fos constitute part of the oral epithelial response to *Candida* hyphal invasion during human infection in vivo. We conclude that MAPK signaling, MKP1, and c-Fos activation play a central role in the discrimination of *C. albicans* yeast and hyphae in epithelial cells, which may have important implications for how the human host responds to and controls *C. albicans* infections in vivo.

## Discussion

Host mechanisms enabling discrimination between commensal and pathogenic organisms are critical in mucosal immune defense and homeostasis. Of particular interest are opportunistic microbes, like *C. albicans*, that can act as either commensals or pathogens under suitably predisposing conditions. We and others have previously shown that hypha formation is crucial for *C. albicans* pathogenicity and induction of proinflammatory responses at mucosal surfaces (Zakikhany et al., 2007; Korting et al., 2003), which results in PMN-dependent TLR4-mediated protection against subsequent fungal infection (Weindl et al., 2007). Given that the key event initiating this protective process appears to be the *C. albicans* hypha-induced proinflammatory response, we sought to identify the epithelial mechanisms that discriminate between the yeast and hyphal forms of *C. albicans*. We report that specific MAPK-based recognition of the filamentous form appears to initiate a danger response mechanism constituting MKP1 and c-Fos activation via a process dependent on fungal burdens. Activation of this mechanism may inform the host of when this normally commensal fungus has become invasive (pathogenic), which may have important implications for mucosal fungal infections in vivo.

Infection of oral epithelial cells with *C. albicans* results in the activation of NF-κB and a biphasic MAPK signaling response, which leads to the induction of a proinflammatory response. Although the activation of NF-κB and MAPK signaling is not unexpected, as these pathways are activated in myeloid cells (Roeder et al., 2004), our study delineates a biphasic MAPK response in epithelial cells that is morphology and burden dependent. Activation of NF-κB and the first MAPK phase appears to be independent of the morphological form and is likely due to the recognition of general fungal cell wall structures. These include chitin, mannans, and β-glucans, as all three moieties activated NF-κB and the first MAPK phase, but not the second MAPK phase. Also, activation of the first MAPK phase does not necessarily result in cytokine induction, as this was readily activated by killed *C. albicans* yeast and hyphae, fungal cell wall agonists, and *S. cerevisiae*, all of which were nonimmunostimulatory. This is in contrast to myeloid cells in which strong inflammatory responses are induced by killed *C. albicans* and fungal agonists (Netea et al., 2006, 2008), indicating that different (or more regulatory) innate response mechanisms exist in epithelial cells. We propose that activation of NF-κB and the first MAPK phase is independent of morphology and constitutes a general epithelial response to inform the host of the presence of fungus.

The second MAPK response involves two key proteins, the MAPK phosphatase MKP1 and the transcription factor c-Fos, and is a specific response to the presence of pathogenic fungus (i.e., *C*. *albicans*). MKP1 activation is mediated via the MEK1/2-ERK1/2 pathway, is associated with filamentation, and is contact dependent but is independent of viability and avidity of adherence. The hypha-associated moieties that induce MKP1 activation are unknown, but cell wall structural components (mannans, chitin, and β-glucans) are unlikely to be the activating factors, as these only activate NF-κB and the first MAPK phase. We propose that MKP1 is activated via cell surface moieties present on filamentous forms of *C. albicans*.

Activation of c-Fos is mediated via the p38 pathway and is also hypha associated, but c-Fos is activated via two independent mechanisms. The first mechanism does not require fungal viability and is probably activated via hypha-associated surface moieties, much like MKP1. The second mechanism is associated with the induction of inflammatory mediators, as c-Fos knockdown significantly reduced cytokine release after *C*. *albicans* infection, and is likely associated with hyphal invasion/penetration and induction of cell damage (as viability promoted c-Fos activation). Our data contrast with that of myeloid cells in which c-Jun rather than c-Fos is thought to play the dominant role in mediating responses to *C. albicans* (Roeder et al., 2004) and further support the notion that epithelial response mechanisms to fungal pathogens are distinct to those of myeloid cells.

Our data suggest that different hypha-associated moieties are required to induce (1) the epithelial MAPK/MKP-1/c-Fos pathway and (2) the cytokine response. This may explain the differences we observed between the two filamentous mutants, in that *Δtup1* might possess one set of surface moieties that activate the MAPK/MKP-1/c-Fos response but lacks another set that results in poor adhesion, lack of invasion, and weak cytokine production. On the other hand, *Δnrg1* may possess both sets of surface moieties, similar to the SC5314 wild-type, and is therefore capable of inducing both the MAPK/MKP-1/c-Fos and the cytokine response. Tup1- and Nrg1-regulated genes have been partially identified (Murad et al., 2001a), and recently, a set of genes was proposed as mediating hyphal virulence independently of morphology (Nobile et al., 2008). Those studies provide several interesting candidates with regard to determining which hyphal moieties might induce the MAPK/MKP-1/c-Fos and cytokine response in epithelial cells.

Although MKP1 and c-Fos activation constitute the second MAPK response, they do not appear to be directly linked, as MKP1 is activated by ERK1/2 and c-Fos by p38. However, the function of MKP1 is to dephosphorylate p38 (and JNK) (Zhao et al., 2006; Chi and Flavell, 2008) in order to stabilize and regulate the overall MAPK network. This is highlighted by an increase in cytokine release (G-CSF, GM-CSF) after MKP1 knockdown or inhibition, as there will be reduced dephosphorylation of p38 (and JNK), which results in an increased effector response (as cytokine induction is primarily mediated via p38 and JNK). In the context of a fungal infection, MKP1 activation will ensure that any immune response to *C. albicans* hyphae is both optimal and tightly controlled while concurrently ensuring a rapid deactivation of potential deleterious p38- and JNK-mediated inflammatory responses when no longer being driven by recognition of hyphae. Given that phosphorylation and stabilization of MKP1 are mediated by ERK1/2 activity, our data imply that ERK1/2 plays an important role in the control and resolution of inflammatory responses by regulating the level of cytokine secretion due to p38 and JNK signaling, rather than by direct effects on cytokine production.

This MAPK/MKP1/c-Fos pathway may be important in vivo because a *C. albicans* strain (529L) that does not form hyphae or activate MKP1, c-Fos, or cytokine production is able to colonize a murine model, whereas a *C. albicans* strain (SC5314) that readily forms hyphae and strongly activates MKP1, c-Fos, and cytokine production is efficiently cleared (Rahman et al., 2007). Likewise, increased levels of MKP1 and c-Fos expression are evident in organotypic models and human biopsies taken from *Candida*-infected individuals. Although we were unable to utilize primary normal oral keratinocytes in our studies, the in vitro data using cell lines appear to be directly applicable to the in vivo situation and suggest that avoidance or activation of this MAPK-based mechanism may be crucial in determining whether a fungal strain colonizes, infects, or is cleared by the host.

Recognition of *C. albicans* by monocytes/macrophages is mediated by several different PRRs, including mannose receptor, TLR2, TLR4, and dectin-1 (Netea et al., 2006). Likewise, in dendritic cells, the initiation of an ERK1/2-c-Fos response is the result of TLR2 and dectin-1 signaling (Agrawal et al., 2003; Dillon et al., 2004, 2006). We show that the MAPK/MKP1/c-Fos signaling mechanism in epithelial cells is unlikely to utilize the same receptors, as knockdown of TLR2 and TLR4 did not affect MKP1 or c-Fos activation or the cytokine profile. Likewise, SYK (the adaptor utilized by dectin-1) was not activated, and β-glucans (the ligand for dectin-1) did not induce cytokine responses. It has been reported that the surface receptor CDw17 may mediate epithelial responses to *Candida* (Li and Dongari-Bagtzoglou, 2009); however, this was against *C. glabrata*, which does not form hyphae. Therefore, it is likely that the MAPK/MKP1/c-Fos system that mediates yeast/hyphal discrimination is through as yet unidentified epithelial PRR(s).

Of major importance is that MKP1 and c-Fos activation is dependent not only on filamentation, but also on fungal burdens, which suggests that a threshold level of stimulation is required prior to full activation of the epithelial response. This may provide a mechanism by which mucosal tissues can remain quiescent in the presence of low fungal burdens (even if hyphae are present) while responding specifically and strongly to damage-inducing hyphae as burdens increase. Therefore, in addition to identifying the switch from the yeast to hyphal form in *C. albicans*, the MAPK/MKP1/c-Fos pathway may also constitute a danger response mechanism informing the host of potentially dangerous levels of this fungal pathogen. We propose that, at low burdens, *C. albicans* avoids activating the MAPK-based danger response pathway, as a threshold level of activation is not reached. As a result, the fungus is regarded as “nondangerous,” thereby permitting successful colonization of human mucosal surfaces without host challenge. One could regard this as *C. albicans* being in the “commensal” state. However, under conditions that permit fungal proliferation, increased fungal burdens together with associated hypha formation activate the MAPK/MKP1/c-Fos pathway to warn the host of the presence of a “dangerous” pathogen. This results in immune activation and secretion of proinflammatory cytokines, which in turn (as we have previously shown), results in PMN recruitment and fungal clearance or reduction of fungal burdens back down below the threshold level of activation and thus a return to the nonactivatory colonization commensal state (Weindl et al., 2007). In the clinical setting, fungal proliferation and immune activation may translate into signs and symptoms of infection, and the cycle of immune activation (based on fungal burdens and hypha formation) followed by subsequent clearance may represent what occurs in patient groups that experience acute *C. albicans* infections.

## Experimental Procedures

### Cell Lines, Reagents, and *Candida* Strains

Experiments were carried out using the TR146 buccal epithelial carcinoma cell line or OKF6-TERT2-immortalized oral cell line (kind gift from Jim Rheinwald). Monolayer cultures were grown in DMEM/10% FBS and experiments carried out in serum-free DMEM. Oral reconstituted human epithelium (5 days) was purchased from SkinEthic Laboratories and used as described (Schaller et al., 2006). ERK (FR180204; 750 nM), JNK (SP600125; 10 μM), p38 (SB203580; 10 μM), and NF-κB (BAY11-7082; 2 μM) inhibitors were from Calbiochem. Antibodies to phospho-p38, -ERK1/2, -JNK, -MKP1, -MEK1/2, and -IκBα and the MEK1/2 (U0126; 20 μM) inhibitor were from Cell Signaling Technologies. Fungal strains included *C. albicans* wild-type SC5314 (Gillum et al., 1984), 529L (in-house clinical strain), *Δtup1* (Braun and Johnson, 1997), *Δnrg1* (Murad et al., 2001b), *Δeed1* (Zakikhany et al., 2007), Δ*efg1/cph1* (Lo et al., 1997), and *S. cerevisiae* (NCPF 3139). *C. albicans* β-glucans microparticles were a gift from David Williams. TLR2 and 4 blocking antibodies were from Invivogen.

### Purification of *C. albicans* Cell Wall Components

Chitin was purified from *C. albicans* cell walls as described (Gow et al., 1980). *O*-linked mannans were extracted using 0.1 M NaOH (room temperature for 20 hr) and *N*-linked mannans with 0.1 M NaOAc (pH 5.2) and 0.5 U endoglycosidase H (overnight at 37°C). Soluble *N*- and *O*-linked mannans were centrifuged, neutralized with either HCl or NaOH, lyophilized, resuspended in deionized water, and extensively purified by ion exchange chromatography using AG50W-X12 and AG4 X4 resins (Bio Rad) until protein negative. Polysaccharides yield was determined by acid hydrolysis with 2 M trifluoroacetic acid (Mora-Montes et al., 2007) and analyzed by high-performance anion-exchange chromatography with pulsed amperometric detection (HPAEC-PAD) (Plaine et al., 2008). Protein content was quantified with the Coomassie assay (Perbio Science) and confirmed endotoxin free (*Limulus* amebocyte lysate assay, Hycult Biotech).

### RNA Extraction and Analysis

RNA was isolated from oral epithelial monolayers and RHE using the Nucleospin II kit (Macherey-Nagel, Thermoscientific) and treated with Turbo DNase (Ambion) to remove genomic DNA. Real-time PCR was performed on cDNA using Jumpstart SYBR green mastermix (Sigma-Aldrich) on a Rotorgene 6000 (Corbett Research). Primers were taken from Primerbank (http://pga.mgh.harvard.edu/primerbank/) (Wang and Seed, 2003) and are listed in the Supplemental Experimental Procedures.

### RNA Interference

Knockdown of gene expression was performed using previously reported siRNAs for MKP1 (Kinney et al., 2008), c-Fos (Chambellan et al., 2009), TLR2 (Opitz et al., 2006), and TLR4 (Weindl et al., 2007). As a control, a nonsilencing siRNA duplex with no homology to known human genes was used as reported previously (Weindl et al., 2007). Transfection of siRNA was performed overnight using HiPerFect reagent (QIAGEN) according to the manufacturer's reverse transfection protocol. qPCR and protein analysis demonstrated that transcript levels for targeted genes were reduced by at least 80% of control levels and maintained this level for at least a further 24 hr.

### Western Blotting

Epithelial cells were lysed using a modified RIPA lysis buffer containing protease (Sigma-Aldrich) and phosphatase (Perbio Science) inhibitors, left on ice for 30 min, and centrifuged for 10 min in a refrigerated microfuge. Supernatants were assayed for total protein using the BCA protein quantitation kit (Perbio Science). 20 μg of protein was separated on 12% NuPAGE Bis:Tris minigels (Invitrogen) before transfer to nitrocellulose membranes (GE Healthcare). After probing with primary and secondary antibodies, membranes were developed using Immobilon chemiluminescent substrate (Millipore) and exposed to ECL film (GE Healthcare).

### Transcription Factor DNA Binding Assay

DNA binding activity of transcription factors was assessed using the TransAM transcription factor ELISA system (Active Motif). In brief, nuclear proteins were isolated from epithelial cells using a nuclear protein extraction kit (Active Motif). Protein concentration was determined, and 5 μg of nuclear extract was assayed in the TransAM system according to the manufacturer's protocol.

### Cytokine Determination

Cytokine levels in cell culture supernatants was determined using the Fluorokine MAP cytokine multiplex kits (R&D Systems), coupled with the Luminex 100 machine according to the manufacturer's protocol. The trimmed median value was used to derive the standard curve and calculate sample concentrations.

### Epithelial Cell Damage Assay

Epithelial cell damage was determined by measuring lactate dehydrogenase (LDH) activity in the culture supernatant using the Cytox 96 Non-Radioactive Cytotoxicity Assay kit (Promega) according to the manufacturer's protocol and using a recombinant porcine LDH (Sigma-Aldrich) to generate a standard curve.

### Adherence Assay

*Candida* strains were incubated with epithelial cell cultures for 30 min and 2 hr. Supernatants were collected (unattached *C. albicans*) and centrifuged at 2500 rpm for 10 min, and the pellets were washed three times with PBS before being plated on Sabouraud's dextrose (SAB) agar and incubated overnight at 37°C. The epithelial cells (with attached *C. albicans*) were washed and then scraped into 1 ml PBS before being plated onto SAB agar plates. Colonies per plate were numerated. Percentage adherence was calculated as: number of adherent colonies (from the epithelial cell pellet) / (number of adherent colonies + number of nonadherent colonies [from the supernatant pellet]) × 100.

### Immunohistochemistry of Oral RHE and Human Biopsies

Human biopsy material (paraffin embedded) surplus to diagnostic requirements was obtained from departmental archives, and its research use under generic opt-out consent was approved by the Guy's Hospital Research Ethics Committee. *C. albicans*-infected oral RHE was fixed in 10% (v/v) formal-saline before being embedded and processed in paraffin wax using standard protocols. For each human biopsy and oral RHE, 5 μm sections were prepared using a Leica RM2055 microtome and silane-coated slides. After dewaxing in xylene, protein expression was determined using rabbit anti-human polyclonal antibodies for MKP1 (Santa Cruz Biotechnology) and c-Fos (Source Bioscience) (1:10 and 1:100, respectively) and counterstained with peroxidase-conjugated goat anti-rabbit secondary IgG antibody, followed by diaminobenzidine (DAB) chromogen detection as per manufacturer's protocol. To visualize *C. albicans*, sections were stained using Periodic Acid Schiff (PAS), counterstained with hematoxylin and examined by light microscopy.

### Statistics

TransAM data were analyzed using a paired t test, and cytokines were analyzed using two-way equal variance t test. In all cases, p < 0.05 was taken to be significant.

## Supplemental Information

Document S1. Supplemental Experimental Procedures and Four Figures
