# Supplementary material for: A Biphasic Innate Immune MAPK Response Discriminates between the Yeast and Hyphal Forms of Candida albicans in Epithelial Cells
Source: Cell Host Microbe. 2010 Sep 16;8(3):225–35. doi: 10.1016/j.chom.2010.08.002 (PMC2991069; doi:10.1016/j.chom.2010.08.002)
Supplement: Document S1. Supplemental Experimental Procedures and Four Figures [file mmc1.pdf]

Cell Host & Microbe, *Volume 8*

Supplemental Information

A Biphasic Innate Immune MAPK Response Discriminates between the Yeast and Hyphal Forms of *Candida albicans* in Epithelial Cells

David L. Moyes, Manohursingh Runglall, Celia Murciano, Chengguo Shen, Deepa Nayar, Selvam Thavaraj, Arinder Kohli, Ayesha Islam, Hector Mora-Montes, Stephen J. Challacombe, and Julian R. Naglik

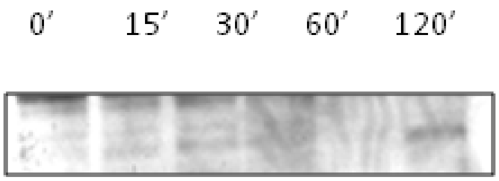

Figure S1: Related to Fig 1. MKP1 phosphorylation in immortalised oral epithelial cells.
Phosphorylation of MKP1 after 2 hours of infection with *C. albicans* in OKF6 epithelial cells. An MOI of 10 was used to infect the cells.

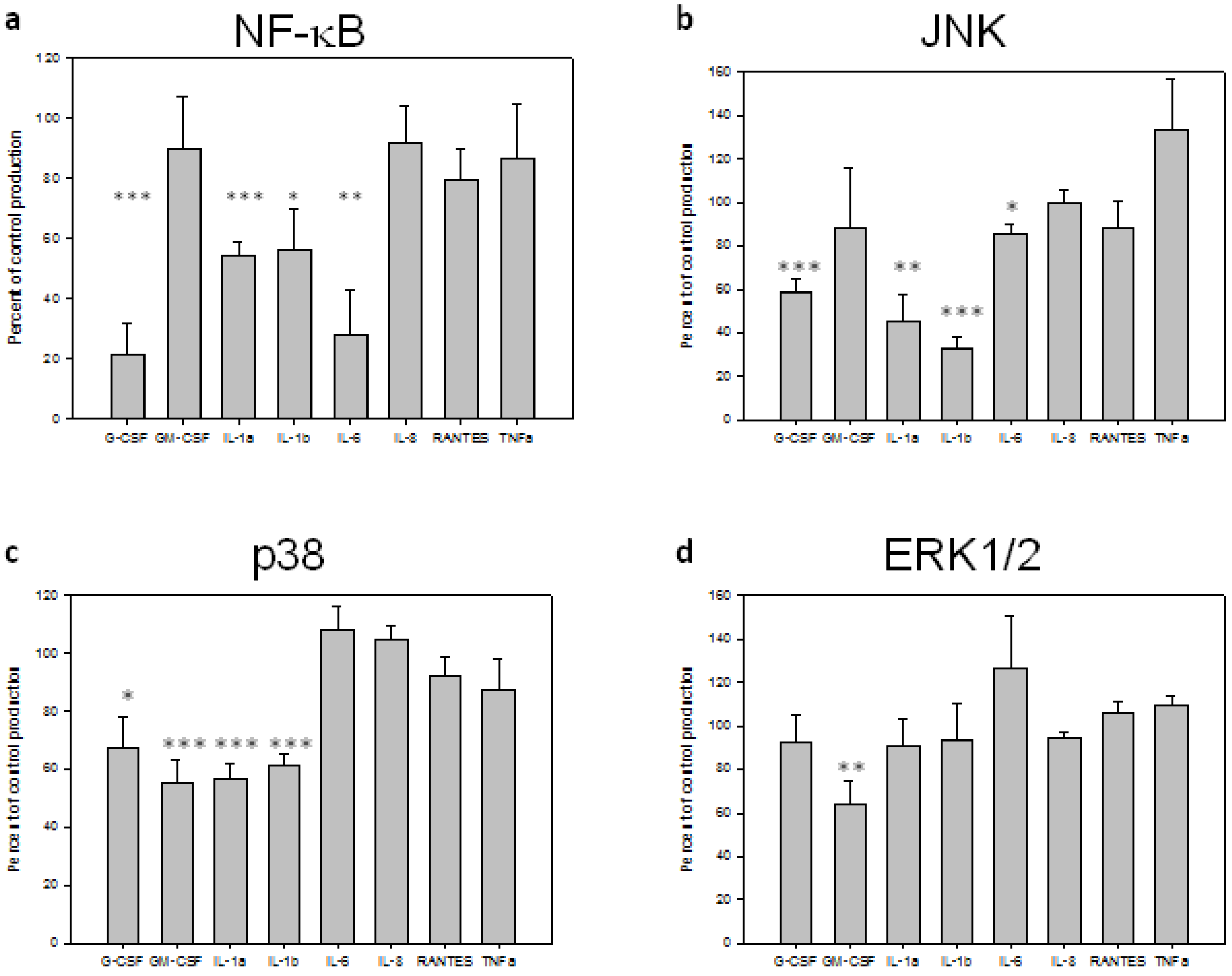
a
NF-κB
b
JNK
c
p38
d
ERK1/2
Percent of control production
G-CSF
GM-CSF
IL-1a
IL-1b
IL-6
IL-8
RANTES
TNFa

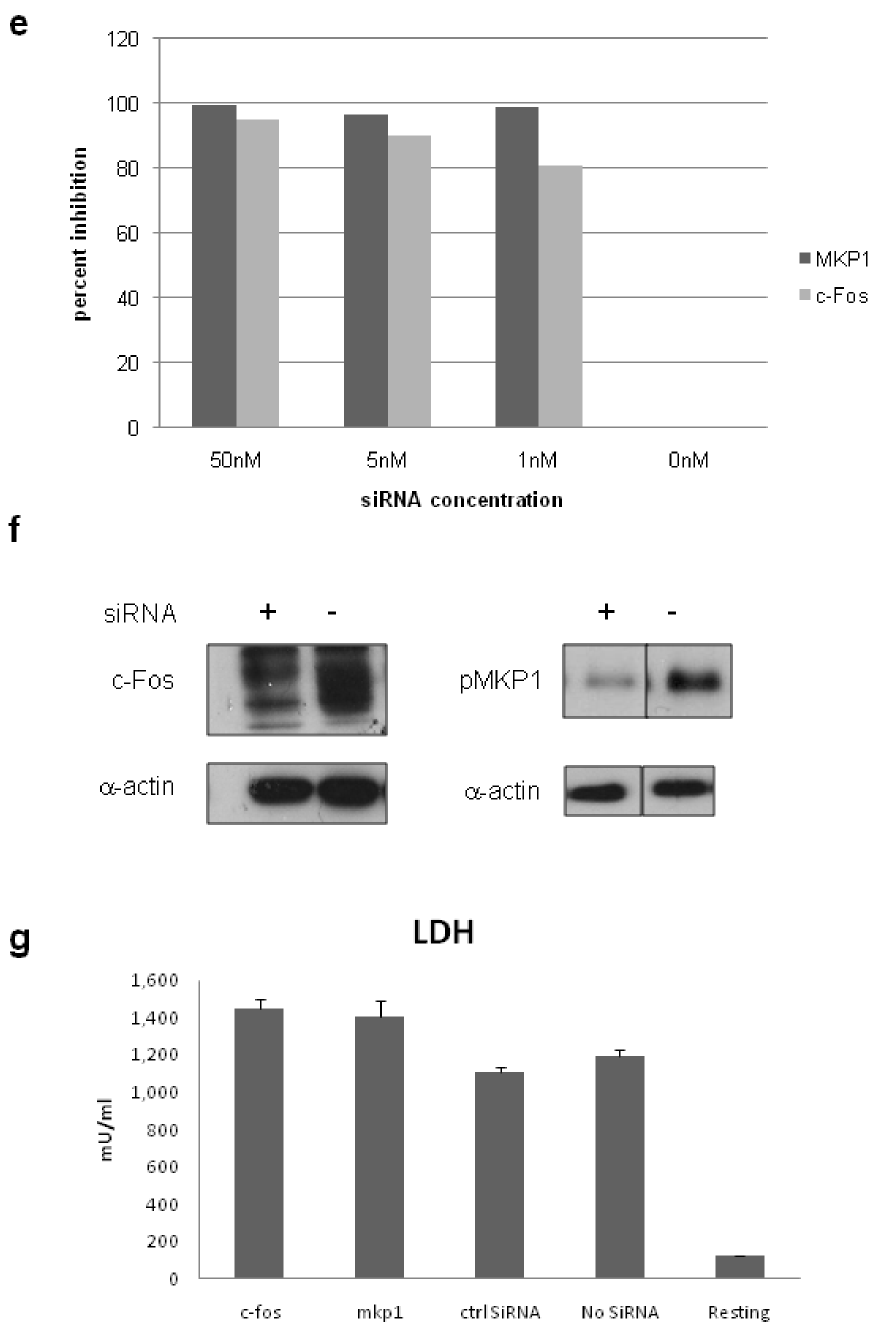

Figure S2: Related to Fig 2. Functional role of NF-κB and MAPK pathways in inducing epithelial effector responses and validation of c-Fos and MKP1 siRNA. Levels of cytokines and gene transcription induced by 24 h infection with *C. albicans* (MOI = 0.01) after pre-treatment for 4 h with (a,) NF-κB inhibitor, BAY11-7082, (b) JNK inhibitor (SP600125), (c) p38 inhibitor (SB203580) or (d) ERK1/2 (FR180204). Well documented IC50 concentrations were used for all inhibitors, which have minimal off-target effects. Results are expressed as percentage of vehicle control. Validation of the MKP1 and c-Fos siRNAs used; (e) TR146 cells were treated with c-Fos and MKP1 siRNA at varying doses for 2 days and the percent of inhibition relative to control siRNA-treated cells for the respective mRNA was ascertained using qPCR. (f) Western blot showing inhibition of protein expression after treatment with 50nM of specific (+) or control (-) siRNA. (g) LDH release by cells with pre-treated with siRNA after infection with *C. albicans*. No significant differences were seen for any of the siRNAs used compared to the control siRNA or the untreated controls. * = $p<0.05$; ** = $p<0.01$; *** = $p<0.001$.

a

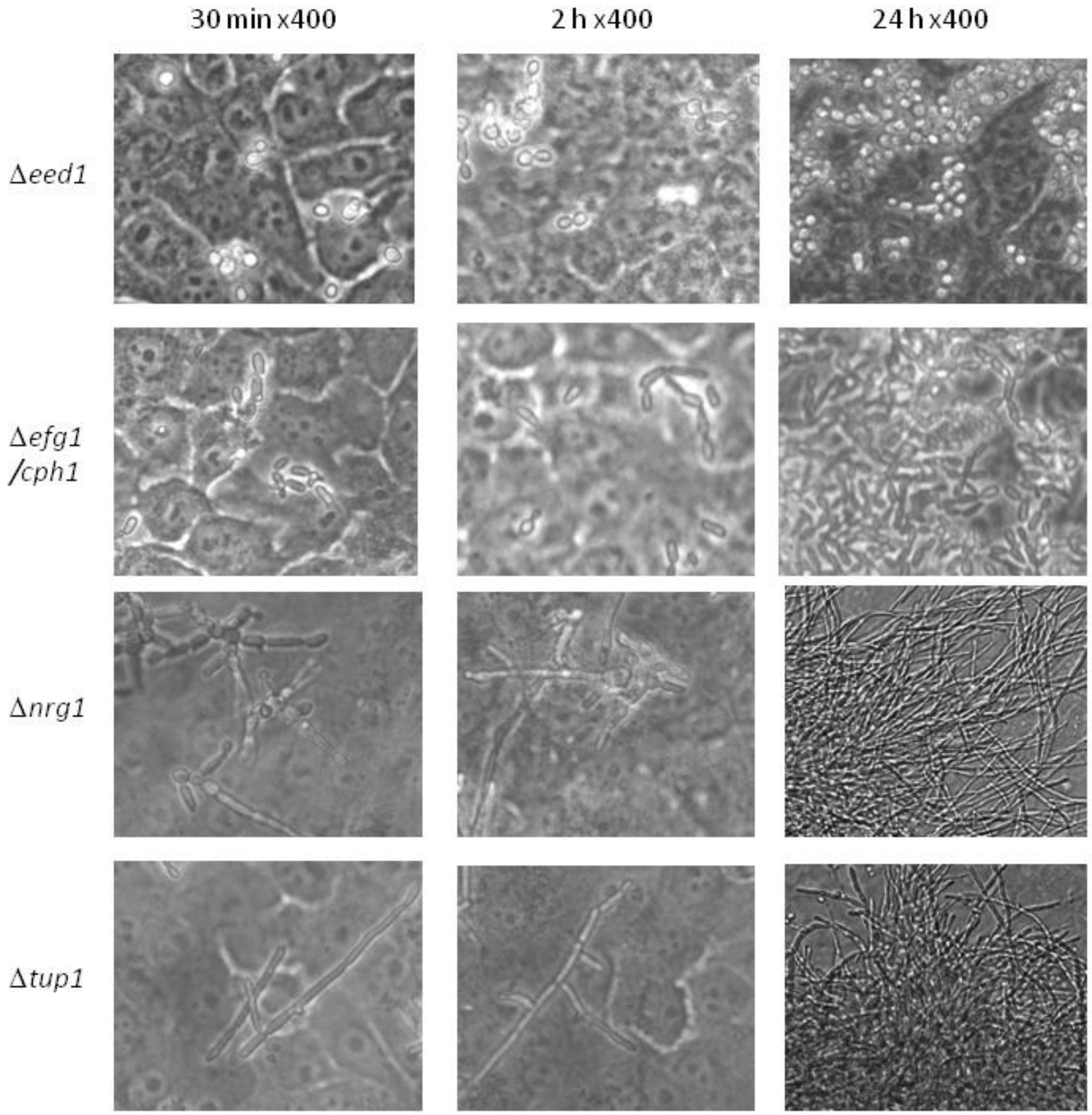
30 min x400
2 h x400
24 h x400
Δeed1
Δefg1 /cph1
Δnrg1
Δtup1

b

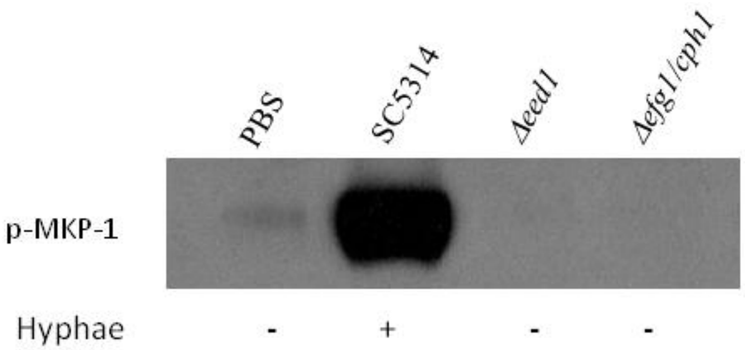
PBS
SC5314
Δeed1
Δefg1/cph1
p-MKP-1
Hyphae
-
+
-
-

c

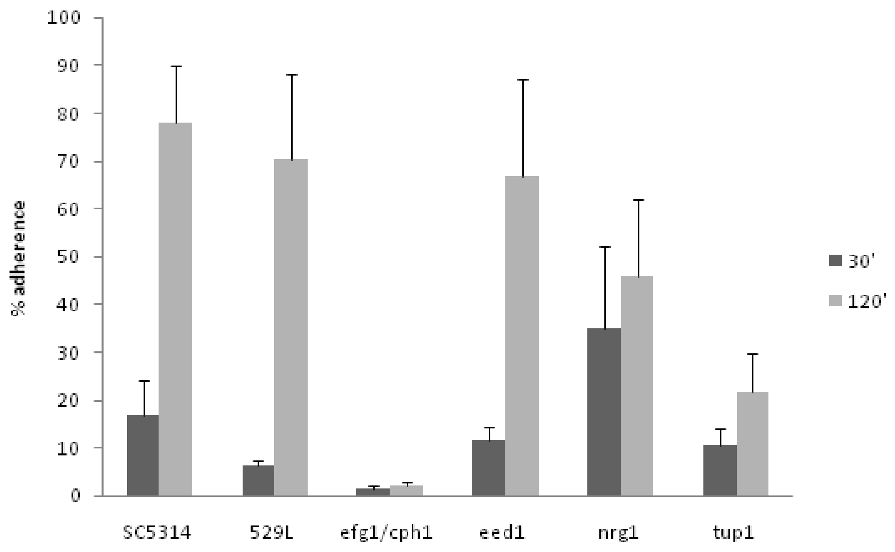

Figure S3: Related to Fig 3. *C. albicans* strain morphology, MKP1 phosphorylation induction and adherence . (a) Morphology of different deletion mutant strains grown on TR146 ECs. As shown, the non-filamentous mutants (Δ*eed1* and Δ*efg1/cph1*) both remain growing in the yeast form, whilst the hyperfilamentous mutants (Δ*tup1* and Δ*nrg1*) both grow exclusively as either hyphae or pseudohyphae. (b) Induction of MKP-1 phosphorylation in oral epithelial cells after infection with wild type (SC5314) and non-filamentous (Δ*eed1*, Δ*efg1/cph1*) *C. albicans* strains for 3 h. An MOI of 10 was used for all infections. Data is representative of 2 experiments. Adherence of different strains and mutants of *C. albicans* to EC monolayers. Data are the mean of 3 independent experiments +/- SD.

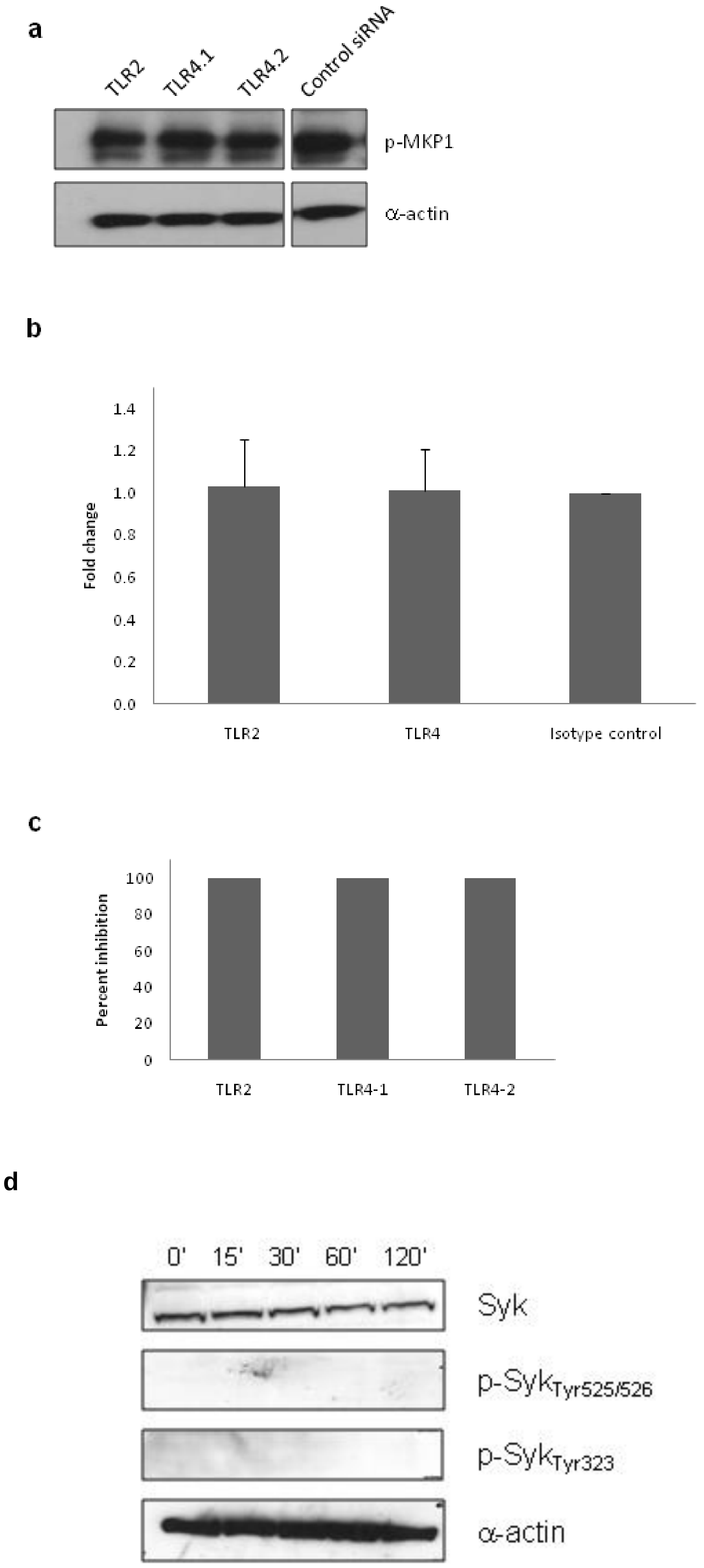

Figure S4: Related to Fig 5. TLR activation in oral epithelial cells by *C. albicans* and siRNA validation; induction of Syk phosphorylation in oral epithelial cells. Lack of inhibition of (a) MKP1 phosphorylation

after 2 hours or (b) c-Fos activation after 3 hours by inhibition of TLR2 or TLR4 using siRNA (a) or blocking antibodies (b). (c) Inhibition effeciences of the different siRNAs after 24 h as measured by qPCR. Each siRNA was used at a concentration of 50nM and efficiences are expressed as percentage inhibitin of control siRNA levels. (d) Lack of phosphorylation of Syk on either tyrosine residue after infection with *C. albicans* at different time points. An MOI of 10 was used for all infections. Data are (a & d) representative of 3 independent experiments or (b) the mean of 4 independent experiments +/- sem.

Supplemental Experimental Procedures

Real-time PCR analysis:

Primers used for qPCR analysis were as follows: YWHAZ foward 5'- ACTTTTGGTACATTGTGGCTTCAA-3', YWHAZ reverse 5'- CCGCCAGGACAAACCAGTAT-3'; MKP1 foward 5'-GGCCCCGAGAACAGACAAA-3', MKP1 reverse 5'- GTGCCCACTTCCATGACCAT-3'; c-Fos foward 5'- CCGCTTGGAGTGTATCAGTCA-3', c-Fos reverse 5'- GGGCAAGGTGGAACAGTTATC-3'; TLR2 foward 5'- GGCCAGCAAATTACCTGTGTG-3', TLR2 reverse 5'- AGGCGGACATCCTGAACCT-3', TLR2 probe 5'- TCCATCCCATGTGCGTGGCC-3'; TLR4 foward 5'-CAGAGTTTCCTGCAATGGATCA-3', TLR4 reverse 5'- GCTTATCTGAAGGTGTTGCACAT-3', TLR4 probe 5'- CGTTCAACTTCCACCAAGAGCTGCCT-3'
